# Global research productivity in the field of discectomy on lumbar disc herniation: A systematic bibliometric analysis

**DOI:** 10.3389/fsurg.2023.1046294

**Published:** 2023-01-31

**Authors:** Wei-Shang Li, Qi Yan, Gao-Yu Li, Wen-Ting Chen, Lin Cong

**Affiliations:** ^1^ Department of Orthopedic Surgery, The First Hospital of China Medical University, Shenyang, China; ^2^ Department of Surgery, University of Texas Health San Antonio, San Antonio, TX, United States; ^3^ Department of Obstetrics and Gynecology, Shengjing Hospital of China Medical University, Shenyang, China; ^4^ Disease Control and Prevention Center of China Railway Shenyang Bureau Group Corporation, Shenyang, China

**Keywords:** discectomy, lumbar disc herniation (LDH), bibliometrics, research hotspots, keywords

## Abstract

**Objective:**

To evaluate the global research productivity in the field of discectomy for lumbar disc herniation (LDH) through bibliometric analysis and mapping knowledge domains.

**Methods:**

A systematic literature search was performed on the Web of Science (WoS), including the Science Citation Index Expanded (SCIE) database and PubMed. The number of publications, countries of publications, journals of publications, total citation frequency, impact factors of journals, and Institutional sources were analyzed by Microsoft Excel 2019, the Online Analysis Platform of Bibliometrics, and VOSviewer. Hotspots were also analyzed and visualized based on VOSviewer.

**Results:**

A total of 2,066 papers were identified. The United States ranked first in the number of total citations (7,970). China ranked first in the number of publications (556, 26.9%), which has surpassed the United States in terms of the number of publications published annually since 2016. Wooridul Spine Hospital published the most papers (43). For journals, *Spine* has published the largest number of papers (289) in this field with the most citation frequencies (6,607). Hotspots could be divided into three clusters: surgery, lumbar disc herniation, and diagnoses. The most recent topic that appeared was symptomatic re-herniation.

**Conclusions:**

The United States is the most significant contributor to the development of discectomy for LDH. The current research focus of discectomy on LDH was the comparison between surgical approaches and evaluation of current minimally invasive discectomy. At present, minimally invasive techniques, such as endoscopic discectomy, cannot completely replace non-endoscopic discectomy (open discectomy and microdiscectomy) through bibliometric analysis and mapping knowledge domains.

## Introduction

Sciatica in adults is mostly caused by lumbar disc herniation (LDH) (1), leading to significant physical disabilities and global health costs (2). Symptomatic LDH is a pathological process that requires surgical intervention after the failure of conservative treatment. In 1934, Mixter and Barr reported the first surgical intervention for symptomatic LDH, namely, open discectomy (3). With the continuous improvement of surgical techniques and instruments, many types of minimally invasive procedures have been developed. Moreover, the application of endoscopy is an innovation for the surgical treatment of symptomatic LDH. Minimally invasive discectomy could be classified into microdiscectomy (MD), microendoscopic discectomy (MED), percutaneous endoscopic lumbar discectomy (PELD), and full-endoscopic discectomy (FED). Both PELD and FED contain two different surgical approaches: transforaminal approach or interlaminar approach (4, 5), whereas these endoscopic procedures do not present a trend of iterative replacement (6–9). At present, the gold standard treatment for symptomatic LDH is still microdiscectomy.

Bibliometrics is a method to assess the trends in global research productivity based on literature databases and literature metrology characteristics. Moreover, its application has become more and more mature in various medical disciplines in recent years, including spinal surgery (10–17). Based on literature data and visualization technology, bibliometrics could be used to display the interaction, crossover, evolution, and derivation among information groups in a certain discipline. Moreover, bibliometrics could provide researchers with a new way to understand the connection between scientific issues and assist them to make superior decisions in either clinical practice or basic medical research. As far as we know, the bibliometric analysis of treatment for LDH has not been reported yet. Therefore, the objective of this study was to evaluate the global research productivity in discectomy on LDH through bibliometric analysis and mapping knowledge domains. The current research status in discectomy on LDH could be explained through the time and spatial distribution of scientific research as well as the analysis of literature topics. Moreover, the future research trend for the treatment of LDH could be predicted reasonably.

## Materials and methods

### Data source and search strategy

A systematic literature search was performed on PubMed, Web of Science (WoS), and the Science Citation Index Expanded (SCIE) database. The search terms were as follows: theme = ((discectomy) AND (lumbar disc herniation or lumbar disk herniation OR LDH)) AND publishing year = (all time) based on MeSH on PubMed. Original articles and reviews were identified.

### Information extraction

Two researchers extracted the data from databases and imported it into Microsoft Excel 2019 independently. The data were imported as follows: number of publications, countries of publications, journals of publications, authors of publications, total citations, impact factors of journals, and institution sources. Disagreements between the two researchers were resolved by consensus after discussion.

### Statistical analysis

The number of publications, contributive countries of publications, contributive journals of publications, total citation frequency, and impact factors of journals and institution sources were analyzed by Microsoft Excel 2019 (Microsoft Corporation, Santa Rosa, CA, United States), which were visualized by GraphPad Prism 8 (GraphPad Software Inc., CA, United States). VOSviewer (Leiden University, Leiden, The Netherlands) is used for visualizing the bibliometric mapping, including literature coupling, co-citation, collaboration, and co-word analysis (18). VOSviewer can also be used to create a map based on text data to mine the themes of articles, which allowed us to analyze the hot spots in certain fields visualized as clusters of masterpieces. The online analysis platform of bibliometrics (http://bibliometric.com/) was also used for analyzing and visualizing the data extracted from databases as a complement.

## Results

### Global publications

**Figure 1:**
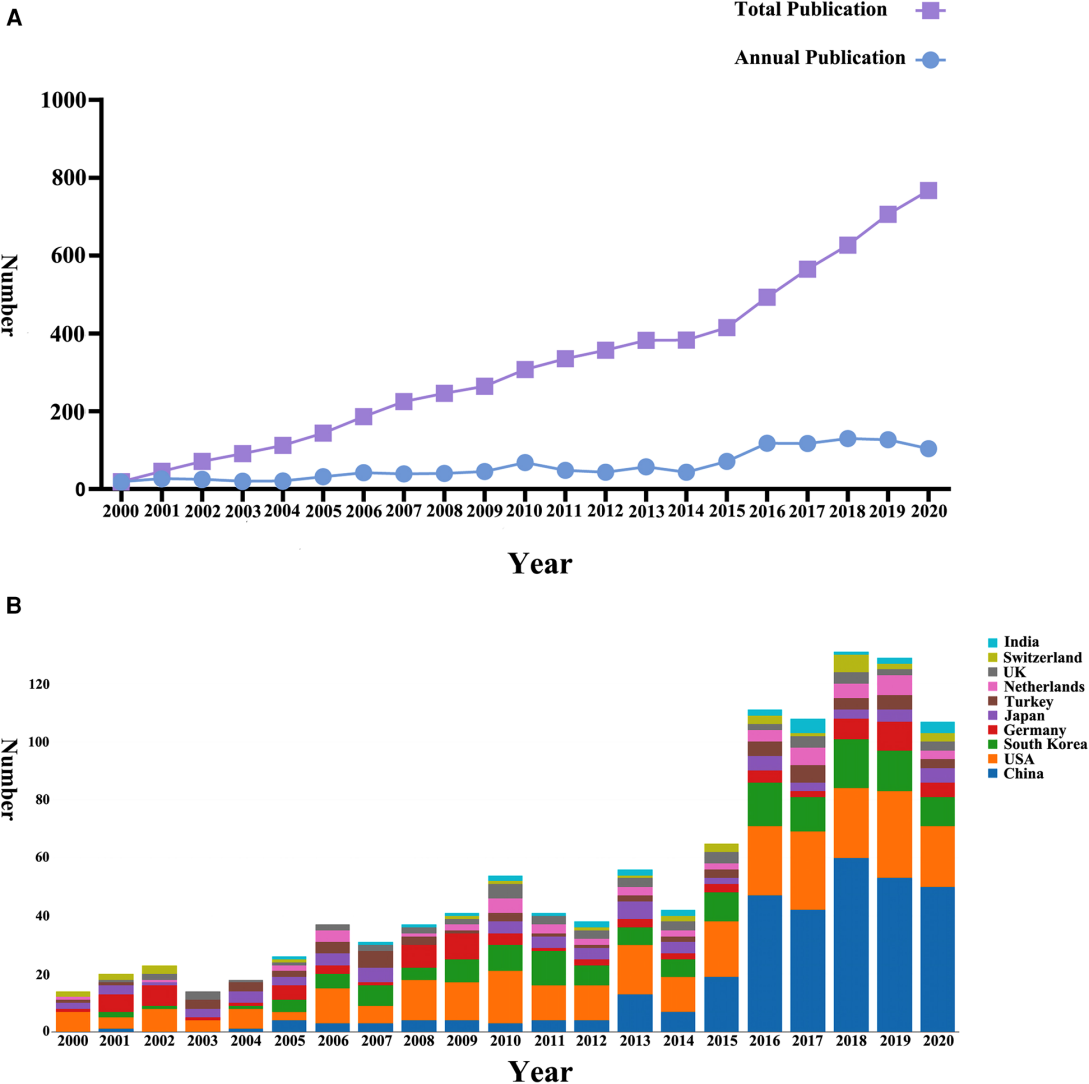
Global trends in research on discectomy from 2000 to 2020. (**A**) The number of publications on discectomy worldwide. (**B**) The number of publications on discectomy worldwide classified by country.

A total of 2,066 papers were identified in this analysis. An overview of global publications in discectomy on LDH over the past 20 years is shown in Figure 1. The total number of publications was on the increase over the years (Figure 1A). The research of China in this field was relatively late, and China has surpassed the United States in terms of the number of publications published annually since 2016 (Figure 1B).

### Highly contributive country, institutions and journals

**Figure 2:**
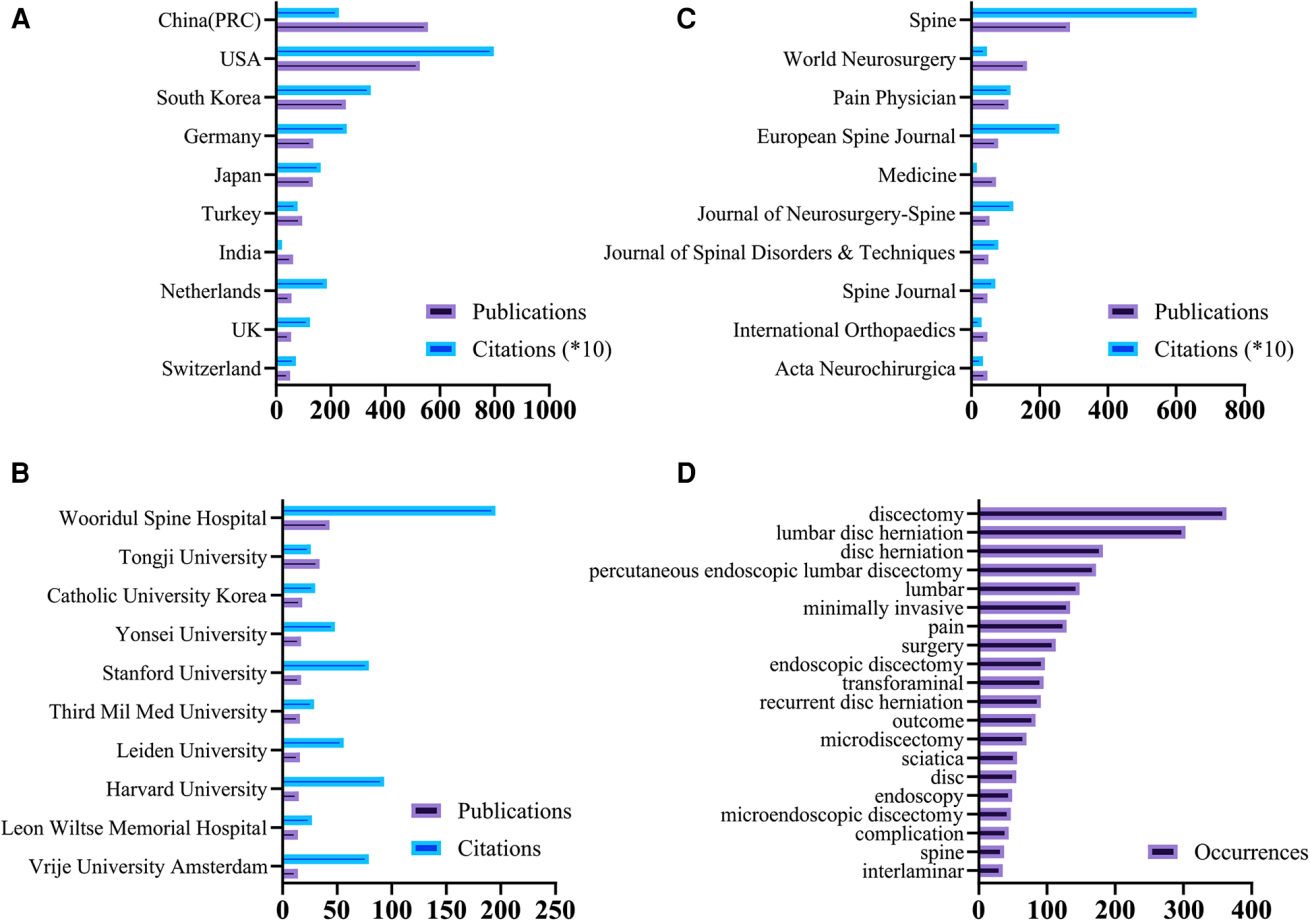
(**A**) The sum number and citation frequency of publications on discectomy from the top 10 contributing countries/regions. (**B**) The sum number and citation frequency of publications on discectomy from the top 10 contributing institutions. (**C**) The sum number and citation frequency of publications on discectomy from the top 10 contributing journals. (**D**) The top 20 keywords of occurrence frequency on discectomy.

The top 10 contributing countries, institutions, and journals by the number of publications are shown in Figure 2 and Table 1. China ranked first in the number of publications (556, 26.9%), followed by the United States (526, 25.5%). The United States ranked first in the number of total citations (7,970), followed by South Korea (3,471). As for institutions, Wooridul Spine Hospital published the most papers (43) and Tongji University ranked second in the number of publications (19). For journals, *Spine* has published the largest number of papers (289) in discectomy on LDH with the most citation frequencies (6,607). *World Neurosurgery* ranked second in the number of publications (163). *European Spine Journal* ranked second in citation frequencies (2586).

**Table 1:** The top 10 countries/regions, institutions, and journals contributing to publications in discectomy.

Country	Publications	Total citations	Institution	Publications	Total citations	Journal	Publications	Total citations
China	556	2,304	Wooridul Spine Hospital	43	195	*Spine*	289	6,607
USA	526	7,970	Tongji University	34	26	*World Neurosurgery*	163	465
South Korea	255	3,471	Catholic University Korea	18	30	*Pain Physician*	109	1,156
Germany	136	2,596	Stanford University	17	79	*European Spine Journal*	78	2,586
Japan	134	1,638	Yonsei University	17	48	*Medicine*	72	162
Turkey	95	789	Leiden University	16	56	*Journal of Neurosurgery-Spine*	53	1,239
India	62	226	Third Mil Med University	16	29	*Journal of Spinal Disorders & Techniques*	50	798
Netherlands	56	1860	Harvard University	15	93	*Acta Neurochirurgica*	47	347
UK	55	1247	Vrije University Amsterdam	14	79	*International Orthopaedics*	47	305
Switzerland	51	728	Leon Wiltse Memorial Hospital	14	27	*Spine Journal*	47	709

### Analysis of keywords and hotspot

**Figure 3:**
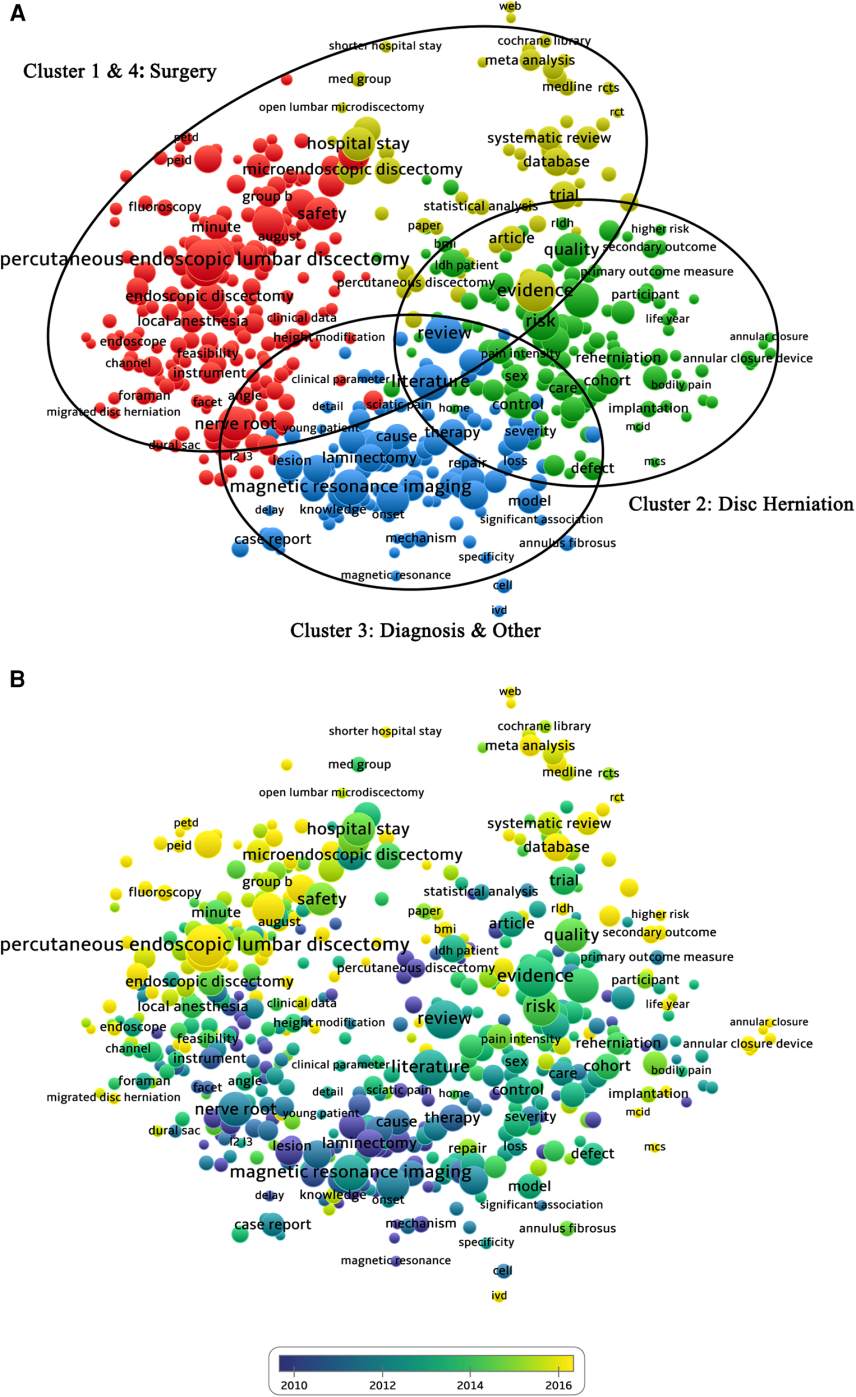
(**A**) Mapping of hotspots in the research on discectomy; the size of the points represents the frequency, and the hotspots are divided into four clusters: surgery (red and yellow clusters in the upper left corner), lumbar disc herniation (green clusters on the right), and diagnoses (blue clusters at the bottom). (**B**) Distribution of hotspots according to the appearance for the average time; hotspots in blue appeared earlier than those in yellow.

The top 20 keywords sorted by frequency of occurrence are shown in Figure 2D. A bibliometric map based on text data Generated by VOSviewer shown the themes of papers in discectomy on LDH classified according to four different colored clusters in Figure 3A. The size of each node represented its weight in the graph. Among the four-color clusters, surgery was the hotspot of papers in the red and yellow clusters on the upper left corner (Clusters 1 and 4); lumbar disc herniation was the hotspot of papers in green clusters on the right (Cluster 2) and those diagnoses of LDH were the hotspot of papers in blue clusters at the bottom (Cluster 3). A chronological distribution of the topics of papers in discectomy on LDH is shown in Figure 3B. According to the average publication year, the most recent topic to appear was symptomatic re-herniation, which occurred 27 times in cluster 2. The earliest topic was chemonucleolysis, which occurred 63 times in cluster 4. The 10 most recent hotspots according to the average publication year are shown in Table 2.

**Table 2:** The 10 most recent hotspots according to the average publication year.

Label	Occurrences	Cluster	Score (avg. pub. year)	Score (avg. citations)
Symptomatic re-herniation	27	2	2018.7407	2.2222
ACD group	20	2	2018.65	1.85
Large annular defect	22	2	2018.6364	3.0455
TELD	29	1	2018.6207	2.2069
Full-endoscopic lumbar discectomy	35	1	2018.1714	3.9143
Annular closure device	49	2	2018.1633	2.551
Shorter operation time	29	1	2018.1379	5.2414
Annular closure	20	2	2018.1	3.05
PTED	65	4	2018.0923	2.7231
ACD	38	2	2018.0526	3.3421

ACD, annular closure device; PTED, percutaneous transforaminal endoscopic discectomy; TELD, transforaminal endoscopic lumbar discectomy.

### Analysis of citation situation

**Figure 4:**
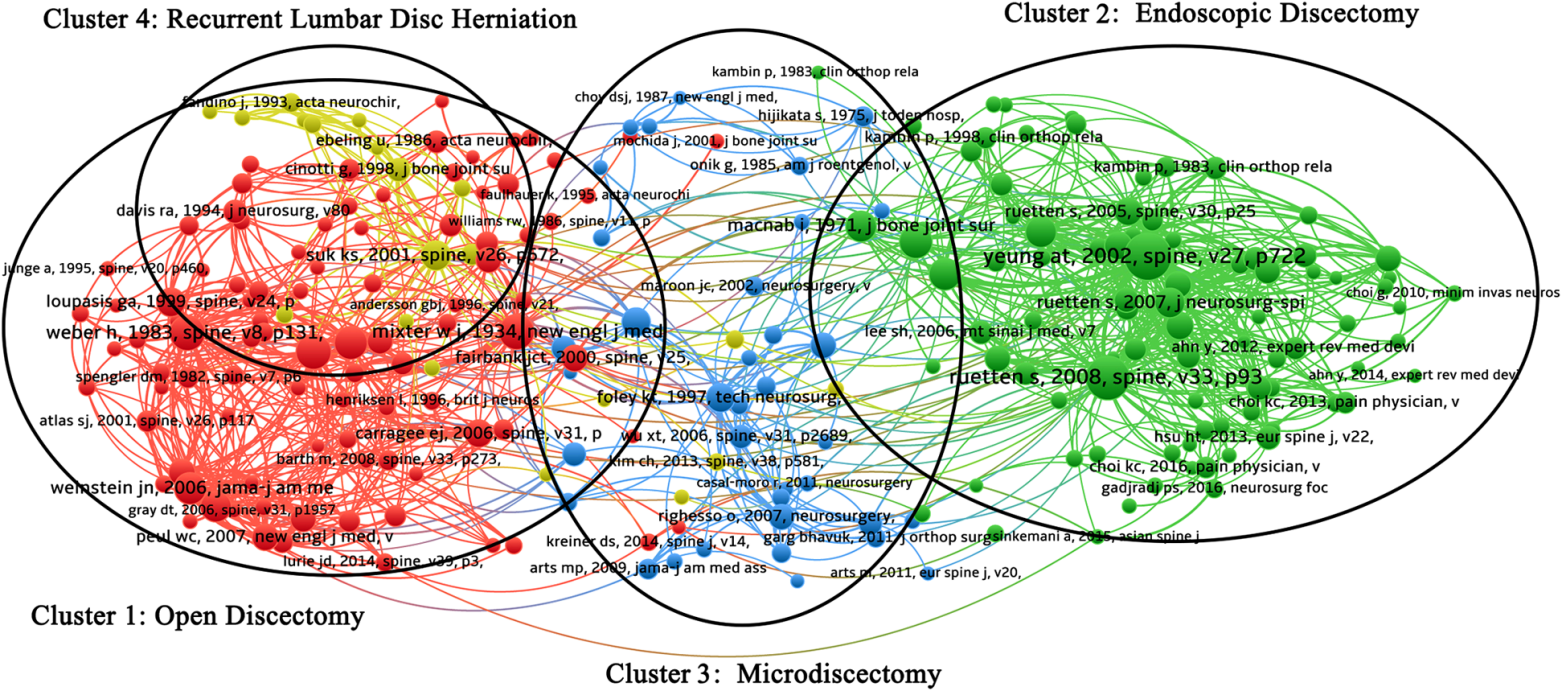
Mapping of citation network related to discectomy. Given the large number of cited references, this study only selected papers cited more than 20 times for analysis and a total 240 papers were included. A line between two points means that both were cited in one paper. Points are divided into four clusters: clusters one (red) are papers related to open lumbar discectomy; clusters two (green) are papers related to endoscopic discectomy; cluster three (blue) contains papers related to microdiscectomy and microendoscopic discectomy; and cluster four (yellow) contains papers related to recurrent lumbar disc herniation.

The citation status of papers in discectomy on LDH is shown in Figure 4, which could be divided into four different-color clusters. The connection represented the degree of connection between each node in this graph. The left red cluster represented the references of open lumbar discectomy (Cluster 1), the middle blue cluster represented the references of microdiscectomy (Cluster 2), and the right green cluster represented the references of endoscopic discectomy (Cluster 3). The yellow cluster in the upper left corner represented references to recurrent lumbar disc herniation (Cluster 4).

## Discussion

With the improvement of patients' requirements for prognosis, discectomy continued to improve as the treatment for symptomatic LDH under the general trend of minimally invasive surgery. By 2020, China ranked the first in the number of publications in the field of discectomy in worldwide (556, 26.9%), followed by the United States (526, 25.5%). However, the citation frequency of Chinese articles was less than that of the United States. In terms of time, China has surpassed the United States in terms of the number of publications published annually since 2016. China started to get involved in the research of discectomy on LDH relatively late, whereas the incidence rate and absolute value of LDH are relatively high in China based on great population, which could provide great number of clinical data. China has considerable experience in the application of various discectomy approaches to LDH. However, none of these surgical approaches to discectomy was invented by the Chinese. China was not as contributive as other countries such as the United States and South Korea, when it came to the creation or renewal of surgical techniques. Similar situations existed in the research institutions. Among the top 10 institutions that contributed the most publications, the number of citations from Chinese institutions was relatively small compared with institutions in other countries. These findings in this study suggest that the United States is the most significant contributor to the development of discectomy in LDH. China developed rapidly in discectomy on LDH but lack of research depth and citation.

For the analysis of the citation situation, Clusters 1–3 were classified according to surgical techniques. Cluster 1 mainly focused on open discectomy, and the representative highly cited articles are published by Weber et al. in *Spine* (118 citations) (20), Carragee et al. in *Journal of Bone and Joint Surgery* (116 citations) (21), and Mixter and Barr in *New England Journal of Medicine* (116 citations) (3). Cluster 2 mainly focused on endoscopic discectomy, and the representative highly cited articles are published by Yeung and Tsou in *Spine* (198 citations) (22), Ruetten et al. in *Spine* (172 citations) (5), and Mayer and Brock in *Journal of Neurosurgery* (101 citations) (4). Cluster 3 focused on microdiscectomy and microendoscopic discectomy, and the representative highly cited articles are published by Caspar et al. in *Advances in Neurosurgery* (81 citations) (23), Maurice et al. in *Techniques in Neurosurgery* (80 citations) (24), and Perez-Cruet et al. in *Neurosurgery* (65 citations) (25). Cluster 4 mainly focused on recurrent lumbar disc herniation, and the representative highly cited articles are published by Suk et al. in *Spine* (87 citations) (26), Cinotti et al. in *Journal of Bone and Joint Surgery* (55 citations) (27), and Swartz and Trost in *Neurosurgical Focus* (47 citations) (19). We found that there were miscellaneous interactions between clusters 1 and 4. The main reason for this finding could be that the researchers found that the surgical intervention was not associated with well-controlled postoperative recurrence in the early stages of discectomy development. Therefore, the papers focusing on recurrent lumbar disc herniation after surgery were performed greatly, which interacted with the papers on open surgery. These papers also prompted researchers and clinicians to improve the approaches of discectomy. With the further development of surgical techniques, the recurrence rate has been controlled and is now in a stable state (6, 28–30). Whereas symptomatic recurrence was still a research hotspot and regarded as one aspect of evaluating surgery in papers at present. Moreover, we found that the highly cited literature studies in each cluster were the first reports or randomized controlled trials of a certain surgical technique. Although systematic reviews and meta-analyses based on the data extracted from these clinical studies were also hotspots, the frequency of citations of these systematic reviews and meta-analyses was lower, which may indicate that the evaluation of different surgical procedures in the field of discectomy on LDH was still not uniform.

In the top 20 keywords sorted by frequency of occurrence, half were associated with surgical techniques and the research of minimally invasive surgery occupied a quite dominant position. In the bibliometric map generated by VOSviewer, clusters 1 and 4 mainly focus on surgical techniques and postoperative clinical outcomes in Figure 3A. The node of “percutaneous endoscopic lumbar discectomy” in cluster 1 ranked first in occurrence. The hotspots of papers in discectomy on LDH were mainly on the evaluation of surgical techniques and the comparison of different surgical techniques. The nodes of “Trial,” “meta-analysis,” and “systemic review” in Cluster 4 indicated that the evaluation of surgical techniques has mostly relied on clinical controlled studies, and different surgical techniques were compared through meta-analysis or systematic review. According to the time-sequence analysis, the early research of discectomy on LDH mainly focused on the pathogenesis of LDH, sciatica, lower back pain, basic surgical procedures, and postoperative failure syndrome. And most recent research focused on minimally invasive surgery and the evaluation of postoperative clinical outcomes. In this bibliometric analysis, the research chronology of discectomy on LDH was consistent with the general law of occurrence and development in modern medicine. Minimally invasive surgery is the inevitable trend of surgery development. However, the real problem is to prove that a newly invented minimally invasive surgery can actually achieve the desired effect, which is equal to microincision. In the current evaluation system, surgical damage is evaluated by a number of indicators, such as operation time, length of hospital stay, intraoperative blood loss, and complications (intraoperative or postoperative). Some studies suggested that the reoperation rate of endoscopic discectomy was higher than that of non-endoscopic discectomy due to the steep learning curve and the limited operative field (22, 31–33). Moreover, the anatomical structure and technique during some endoscopic discectomy (such as percutaneous endoscopic transforaminal discectomy) are not similar to traditional non-endoscopic discectomy. The surgeons not adapt to this approach very quickly, which makes the introduction of the endoscope relatively difficult in the early stages. In addition, for complicated situations such as malformations and degeneration, non-endoscopic discectomy can achieve better clinical results and ensure safety. From the findings in this study, the current research focus of discectomy on LDH was the comparison between surgical approaches and evaluation of current minimally invasive discectomy. At present, minimally invasive techniques such as endoscopic discectomy cannot completely replace non-endoscopic discectomy (open discectomy and microdiscectomy) through bibliometric analysis and mapping knowledge domains.

A consensus has been reached that the operation for re-herniation is associated with more cost and is less effective (34–37). Hence, reducing the re-herniation rate has always been one of the topics in the field of discectomy. Studies suggested that the rate of re-herniation after lumbar discectomy has decreased to approximately 3% in 2017 (38), whereas some recent studies reported that patients with large postoperative annular defects (≥6 mm width) had a 2.5-fold higher rate of recurrence, compared with patients who had small annular defects (<6 mm width) (39, 40). In recent years, a method for repairing an annular defect to control the re-herniation rate has been proposed, which includes the implantation of an annular closure device (ACD). Through bibliometric analysis and mapping knowledge domains in this study, the recent appeared topic included symptomatic re-herniation, large annular defect and annular closure device shown in Figure 3B and Table 2. By combining bibliometrics analysis with clinical practice, we predict that one of the research hotspots in the future could be the control of recurrence rate and the solution of symptomatic re-herniation after lumbar discectomy.

The objective of this study was to evaluate the global research productivity in the field of discectomy on LDH through bibliometric analysis and mapping knowledge domains. Previous published studies have investigated the quantity and quality of articles in the field of full-endoscopic spine surgery (15). The advantage of this study was that we focused on a specific surgical intervention, namely, discectomy for lumber disc herniation, which narrowed the scope of research and improved its depth, but there were still several limitations in this study. First, the databases that we indicated mainly contain publications in English, which lacked non-English literature. Second, differences may exist between the real research situation and the bibliometric analysis findings because of the calculation load of the algorithm. Some recently published high-quality papers were removed because the number of citations did not reach the set threshold. Third, some of the latest articles may not be included in the databases yet.

## Conclusions

The United States is the most significant contributor to the development of discectomy for LDH. Some countries developed rapidly in the number of publications but lacked research depth and citations, such as China. The current research focus of discectomy on LDH was the comparison between surgical approaches and evaluation of current minimally invasive discectomy. At present, minimally invasive techniques such as endoscopic discectomy cannot completely replace non-endoscopic discectomy (open discectomy and microdiscectomy) through bibliometric analysis and mapping knowledge domains. One of the research hotspots in the future is the control of the recurrence rate and the solution of symptomatic re-herniation after lumbar discectomy.

## Data Availability

The original contributions presented in the study are included in the article/Supplementary Material, further inquiries can be directed to the corresponding author.
